# Vibration therapy as an intervention for enhancing trochanteric hip fracture healing in elderly patients: a randomized double-blinded, placebo-controlled clinical trial

**DOI:** 10.1186/s13063-021-05844-y

**Published:** 2021-12-04

**Authors:** Ronald Man Yeung Wong, Simon Kwoon Ho Chow, Ning Tang, Yik Lok Chung, James Griffith, Wing Hong Liu, Raymond Wai Kit Ng, Chi Yin Tso, Wing Hoi Cheung

**Affiliations:** 1 Department of Orthopaedics & Traumatology, The Chinese University of Hong Kong, Hong Kong, China; 2 Department of Orthopaedics & Traumatology, Prince of Wales Hospital, Hospital Authority, Hong Kong, China; 3 Department of Imaging and Interventional Radiology, The Chinese University of Hong Kong, Hong Kong, China

**Keywords:** Hip fractures, Vibration treatment, Osteoporosis, Trochanteric fracture, Randomized controlled trial

## Abstract

**Background:**

There are more than 300,000 hip fractures yearly in the USA with mortality rates of 20% within 1 year. The treatment of osteoporotic fractures is a major challenge as bone quality is poor, and healing is expected to delay due to the impaired healing properties with respect to bone formation, angiogenesis, and mineralization. Enhancement of osteoporotic fracture healing and function is therefore critical as a major goal in modern fracture management. Previous pre-clinical studies have shown that low-magnitude high-frequency vibration (LMHFV) accelerates osteoporotic fracture healing. The objective of this study is to investigate the effect of LMHFV on accelerating trochanteric hip fracture healing and functional recovery.

**Methods:**

This is a randomized, double-blinded, placebo-controlled clinical trial to evaluate the effect of LMHFV in accelerating trochanteric hip fracture healing. All fractures undergo cephalomedullary nail fixation. The primary outcome of this study is time to fracture healing by X-ray. Computed tomography (CT) and dual-energy X-ray absorptiometry (DXA) will also be performed. Blood circulation at the fracture site will be assessed by dynamic perfusion magnetic resonance (MR). Clinical results include functional recovery by muscle strength, timed up and go test (TUG), quality of life questionnaire (SF-36), balancing, falls, and mortality.

**Discussion:**

Previous animal studies have demonstrated LMHFV to improve both normal and osteoporotic fracture healing by accelerating callus formation and mineralization. The mechanical stimulation stimulates angiogenesis by significantly enhancing vascular volume and blood flow velocity. This is the first study to translate LMHFV to enhancing hip fracture healing clinically. Positive results would provide a huge impact in the recovery of hip fracture patients and save healthcare costs.

**Trial registration:**

Clinicaltrials.gov NCT04063891. Registered on August 21, 2019

**Supplementary Information:**

The online version contains supplementary material available at 10.1186/s13063-021-05844-y.

## Background

Hip fractures are currently ranked as the top 10 causes of disability [1]. The leading cause is due to osteoporosis and the lifetime fracture risk of osteoporotic patients reaches as high as 40% [2]. There are approximately 2.5 million osteoporotic fractures each year in the United States (US), with costs estimated at 15 billion USD in 2010 [3]. In fact, 300,000 hip fractures occur yearly in the US with mortality rates of 20% within 1 year [4]. Furthermore, in the United Kingdom (UK), approximately 80,000 hip fractures are treated yearly with direct costs of 2 billion pounds and mortality of 30% at 1 year [5]. The World Health Organization (WHO) predicts that the aging population will continue to rise over the next 25 years, and therefore, the socioeconomic burden is expected to grow rapidly.

The treatment of osteoporotic fractures is a major challenge as bone quality is poor, and healing is expected to delay due to the impaired healing properties with respect to bone formation, angiogenesis, and mineralization [6–8]. Failure to unite results in pain, weakness, reduced mobility, and surgical fixation failure, and these complications are most common in elderly patients, which can lead to serious detrimental effects to overall health status. Furthermore, due to prolonged recovery, muscle function often deteriorates as well. Enhancement of osteoporotic fracture healing and function is therefore critical as a major goal in modern fracture management.

Low-magnitude high-frequency vibration (LMHFV) is a promising biophysical intervention that provides non-invasive, systemic mechanical stimulation. Previous animal studies have demonstrated LMHFV to improve both normal and osteoporotic fracture healing by accelerating callus formation and mineralization [6, 7, 9, 10]. The mechanical stimulation stimulates angiogenesis by significantly enhancing vascular volume and blood flow velocity [7]. Further investigating into the molecular pathways, we have shown LMHFV to enhance healing from the early inflammation stage to the late phases of remodeling [7, 10–13]. For the clinically relevant animal model of metaphyseal fracture healing [14], fibrinolysis was also enhanced with LMHFV to accelerate this process [15]. Improved healing was also seen with enhanced ultrastructural and functional changes of osteocytes [16]. Clinical trials have also shown positive improvements in reaction time, movement velocity, balancing ability, and quadriceps muscle strength in healthy subjects [17], which were retained 1 year after cessation of LMHFV treatment [18].

Justified with the positive results from previous pre-clinical studies, the aim is translate the use of LMHFV to enhancing hip fracture healing in clinical patients. A randomized double-blinded, placebo-controlled clinical trial is to be conducted to assess the efficacy of LMHFV in accelerating trochanteric hip fracture healing. The primary outcome is fracture healing. The secondary outcomes are densitometry, circulation, function, pain, quality of life, falls, and mortality.

## Methods

### Study design and standardization of surgical management

**Fig. 1:**
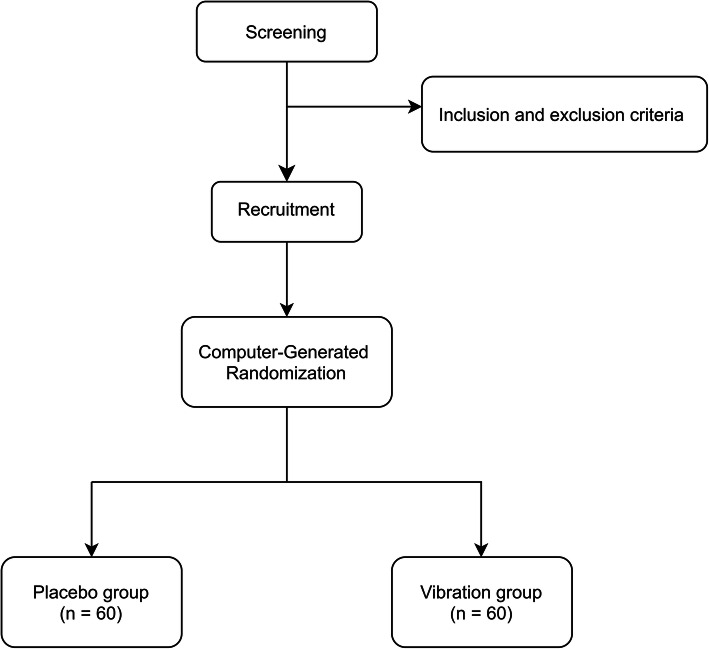
A flowchart of the study design. The Consolidated Standards of Reporting Trials (CONSORT) checklist is provided as Additional file 1; the Standard Protocol Items: Recommendations for Interventional Trials (SPIRIT) checklist is presented in Additional file 2

This is a randomized, double-blinded, placebo-controlled clinical trial to evaluate the effect of LMHFV (VH-001 exercise platform; V-health Limited, Hong Kong) on accelerating trochanteric hip fracture healing (Fig. 1). Trochanteric hip fracture patients admitted to the Orthopaedics and Traumatology ward are recruited, which are from the Prince of Wales Hospital, affiliated with the Chinese University of Hong Kong. All patients would be screened for the inclusion and exclusion criteria to meet eligibility for the study. There are more than 400 hip fractures admitted to our unit each year. Closed reduction and cephalomedullary nail fixation (Gamma3 nail, Stryker, USA) [19] will be performed for all patients within 48 h. Orthogeriatric co-care is provided for each patient to assess and treat any medical complications before operation [20]. Prophylactic intravenous antibiotic with cefazolin 1 g is given before surgical incision and continued for 3 doses after every 8 h. Postoperatively, pressure bandage will be given until mobilization. Adequate analgesics will be given. Full weight bearing with physiotherapy is started as soon as condition allows.

### Inclusion criteria

The inclusion criteria are as follows:

1. Elderly male or females aged 65 years or older
2. Unilateral trochanteric hip fractures (AO classification A1-A3)
3. Due to unintentional fall
4. Fractures fixed with cephalomedullary nail (Gamma nail, Stryker)
5. Willing and able to comply with study protocol

### Exclusion criteria

Exclusion criteria are as follows:

1. Open fracture
2. Bilateral lower limb fractures
3. Patient with multiple injuries affecting balance/standing on platform
4. Pathological fractures e.g., tumor, infection.
5. History of medication or disease affecting bone metabolism such as hypo/hyperthyroidism., etc.

### Malignancy

6. Chairbound or bedbound
7. Cognitive problems, e.g., severe dementia.

### Drop-out criteria

Drop-out criteria are as follows:

1. Refusal to participate
2. Default follow-up
3. Reported to be unsafe for treatment

### Sample size

In this study, time to radiologic healing is the primary objective. This is a well-accepted quantitative approach to evaluate fracture union, as in our previous study [21]. We expect 20% acceleration of radiologic healing time (our animal data confirms 30% acceleration [9]). A sample size of 100 will detect a significant difference using ANOVA with 80% power and 0.05 significance level (PASS 11.0, NCSS, LLC, Utah, USA). Our previous clinical trial [17] confirmed that compliance of vibration treatment was very good with normal subjects at 66% on average [17]. We conservatively increase the sample size to 120, assuming 15% dropout rate.

### Randomization and blinding

Patients will be actively screened and recruited from the Department of Orthopaedics and Traumatology, Prince of Wales Hospital, Hospital Authority, Hong Kong, based on inclusion and exclusion criteria. According to our previously established protocol [22], a computer-generated set of random allocations are sealed in consecutively numbered opaque envelopes by an independent central technician. Once consent is obtained, the patient is randomized and assigned into either vibration or placebo group by opening the next sealed envelope by a specific staff member. Patients and investigators are fully blinded until the completion of treatment. Outcome assessors and statistician are blinded.

Patient demographics will be recorded and confirmed with the Clinical Management System (CMS), Hospital Authority, which is the central electronic database for public hospitals in Hong Kong [23]. Before signing the consent form, each patient will be explained the objectives, benefits, and risks of the study and their rights and responsibilities, as well as privacy and confidentiality information. An information sheet will also be distributed.

### Interventions

Patients will be randomly allocated to placebo or vibration group [22]. Vibration is treated with LMHFV (V-health Ltd, Hong Kong) at 35 Hz, 0.3 g (*g* = gravitational acceleration) for 20 min/day, 5 times/week [17], while the placebo group will have sham treatment to stand on the LMHFV platform for 20 min/day, 5 times/week. Sound emitted by machine masks the noise an active machine makes [24]. Treatment is started at 3 days post-operation for a total of 14 days (protocol change from 6 months due to COVID-19 for patient feasibility). Patients will be followed up to 6 months.

## Outcome assessments

### Plain radiographs

Anteroposterior and lateral X-ray (DRX-Evolution, Carestream Health, Rochester, NY, USA) of fracture site will be performed at baseline, 6, 12, and 26 weeks post-operation. Films will be assessed by two blinded surgeons independently, to note healing in the AP or lateral film [21]. Fracture healing is defined based on the Radiographic Union Score of Hip (RUSH), which assesses callus formation and visibility of fracture line at 4 cortices [25].

### Computed tomography (CT)

CT (SOMATOM Drive, Siemens, Erlangen, Germany) scan will be performed at the region of interest 5 cm proximal and distal to the fracture site [26], at baseline, 6, and 12 weeks post-operation. Films will be assessed by a blinded radiologist.

### Clinical monitoring

Standard postoperative program for all in-patients, including vital sign monitoring, wound condition, neurovascular status, pain, analgesic, and mobilization.

### Densitometry status

Bone mineral density (BMD) and bone mineral content (BMC) will be measured at baseline, 6, 12, and 26 weeks post-operation using DXA (Horizon DXA System, Hologic Inc., USA) to monitor changes in bone quality at the fracture site [18].

### Dynamic perfusion MRI

Blood circulation at fracture site will be measured at baseline and 6 weeks post-operation with dynamic contrast-enhanced MR imaging (Intera NT; Philips Medical Systems, Best, Netherlands) with maximum gradient strength of 30 mT/m as in our previously established protocol [27, 28].

### Functional and quality of life outcomes

At baseline, 2, 6, 12, and 26 weeks post-operation, all patients will be assessed with the following functional tests:

### Verbal descriptor scale

Verbal descriptor scale will be used to assess different levels of pain intensity, which has good reliability and validity in elderly patients [29].

### Quality of Life Short Form-36 (SF-36)

SF-36 is a well-validated functional status questionnaire to measure health-related quality of life in eight domains, including physical functioning, health perception, etc. A higher score indicates better function [17].

### Quadriceps muscle strength

Quadriceps muscle strength will be measured on the affected limb with an isometric dynamometer (Baseline, Genova, Italy). Subject will sit on a chair with both feet above ground, while raising the affected leg 45° forwards. The dynamometer will be placed above the ankle and the subject will push the leg forward with maximum force (N). Measurements will be repeated three times and the maximum value will be used for evaluation [17].

### Balancing ability

To assess balancing ability, the Biodex Balance System SD (Biodex Medical Systems Inc., Shirley, NY, USA) is used to measure the static and dynamic ability of patients to maintain center of balance. The score generated by the machine assesses the deviation from the center via an Overall Stability Index (OSI), Anterior/Posterior Stability Index (APSI), and Medial/Lateral Stability Index (MLSI), which have been shown to be reliable tools for objective assessment of postural stability in several studies in elderly patients [22].

### Times Up and Go (TUG) test

The TUG test will be used to test basic mobility skills, which is a useful predictor of risk of falls. The patient will stand from a chair, walk 3 m, and travel back and sit back on the chair. The time in seconds (s) is recorded [22].

### Falls

Mortality will be measured at 1 month, 3 months, and 1 year via a fall calendar, which is well established in clinical trials for elderly patients [17].

### Mortality

Mortality will be measured at 1 month, 3 months, and 6 months [30]. Mortality is documented from the Clinical Management System (CMS) from Hospital Authority, a central public computerized system for patients in Hong Kong [23].

### Compliance assessment

A smart card is also given to each participant to record compliance to placebo and LMHFV [17].

### Adverse event handling

Currently, there is no report of adverse event in the use of LMHFV. In case of unusual conditions including intolerable pain, LMHFV treatment will be terminated and clinicians will follow-up the patient. Any adverse events or problems during the study will be verified and documented by independent staff members.

### Data collection, monitoring, and management

The research assistant will be trained to ensure accuracy of outcome assessments and data collection. The ethics committee will oversee and audit any issues raised and corresponding measures will be taken if necessary. Subjects not willing to continue can withdraw at any time. The study will comply with the good clinical practice guidelines. Each patient will be assigned an identification code. The patient identification code list and database will be safeguarded.

### Statistics

All results will be expressed in mean ± standard deviation. Statistical analysis will be performed using SPSS (IBM, NY, USA). One-way (for between-group differences in over-time changes), two-way (for interaction effect among time and group), and group repeated measures ANOVA with post-hoc Bonferroni analysis, as well as independent/paired *t* tests will be performed. Significance level is set at *p* < 0.05 (2-tailed).

### Dissemination plans

We will disseminate the trial results to healthcare professionals, the public, and other relevant groups as soon as results are available (Appendix).

## Discussion

This is the first double-blinded, placebo-controlled trial to investigate the efficacy of LMHFV in accelerating trochanteric hip fracture healing. With the current aging population and increasing number of hip fractures, strategies to enhance healing and functional recovery are crucial. LMHFV is a non-invasive treatment, which is easily acceptable for elderlies and has proven benefits for physical function in a previously conducted randomized controlled trial of 710 healthy, active, and independent postmenopausal women [17]. Benefits of LMHFV for balancing ability, muscle strength, and risk of falling were retained 1 year after cessation of the treatment [18]. No serious side effects were noted. Numerous animal studies have also shown LMHFV to significantly improve normal and osteoporotic fracture healing with increased callus formation, angiogenesis, and mineralization [6, 7, 9, 10]. However, clinical translation in hip fractures remains to be proven.

Current Fracture Liaison Services (FLS) aims to close the osteoporosis care gap and prevent secondary fractures that can lead to high morbidity and mortality [20]. However, the presence of sarcopenia often co-exists with osteoporosis leading to the geriatric syndrome “osteosarcopenia” [31]. The prevalence of sarcopenia also reaches up to 95% in males and 64% in females in osteoporotic fracture patients [32]. It has been postulated that most patients develop muscle deterioration during immobilization from a fracture. Therefore, the incorporation of effective strategies to accelerate fracture healing for early pain-free mobilization has been advocated.

The risk of an imminent fracture is also high after an initial osteoporotic fracture. In fact, more than 50% of secondary fractures occur during the first 2 years [33]. Therefore, this window of opportunity is also a golden period for intervention. Positive results of LMHFV in enhancing fracture healing and functional outcome would have a significant impact for hip fracture patients by decreasing disability. More importantly, the incorporation of LMHFV into FLS can potentially lead to a significant decrease in health care costs.

Enrolment in this trial is planned to begin in January 2021. Completion is expected to take 24 months. We speculate that positive results would allow the incorporation of LMHFV into hip fracture multidisciplinary rehabilitation programs.

The completion of the trial is expected to take 24 months.

## Trial status

The trial was initially planned to start on January 2020. However, due to the COVID-19 situation, the trial had started later in September 2021, and the protocol on vibration period and assessment time points had changes compared to the original plan for feasibility of the study.

### Supplementary Information

**Additional file 1.** Consolidated Standards of Reporting Trials (CONSORT).

**Additional file 2.** Standard Protocol Items: Recommendations for Interventional Trials (SPIRIT) checklist.

## Appendix

**Table 1:** Study plan

Time frame	Key milestones
1–12 months	Enrolment of patients
1–12 months	Low-magnitude high-frequency vibration and placebo treatments
1–18 months	Outcome assessments
18–24 months	Conference
18–24 months	Manuscript preparations
